# Highly Cited Papers at the Spanish Domestic Level

**DOI:** 10.3389/frma.2021.651991

**Published:** 2021-04-22

**Authors:** Carlos García-Zorita, Sergio Marugán, Daniela De Filippo, Elías Sanz-Casado

**Affiliations:** ^1^Department of Library and Information Science, Carlos III University of Madrid (UC3M), Getafe, Spain; ^2^INAECU Research Institute for Higher Education and Science Autonomous University of Madrid (UAM) Carlos III University of Madrid (UC3M), Madrid, Spain

**Keywords:** highly cited papers, Spanish universities, visibility indicators, impact indicators, higher education institutions

## Abstract

This paper presents a methodological proposal based on the identification of highly cited papers (HCPs) at domestic-level in the Spanish Public University System (SUPE), in order to find the most outstanding publications in the local context. The principal aim is to detect different activity and impact profiles among Spanish universities and differentiate those institutions that play a more significant role. To determine which and how many are the highly cited papers at the domestic level (HCP-DL) collected in the Web of Science, three citation thresholds (1, 5, and 10%) were established. Thematic classification in Incites/Essential Science Indicators areas is used. The results show a preponderance of HCPs in the field of Space Science, while the polytechnic universities have high visibility in the Computer Science area. It has been observed that the presence of HCPs in a given area is involved with universities specialized in teaching and research activities. In absolute terms, the big non-specialized universities are major producers of HCPs and hold the leading positions in our results. However, when efficiency is analyzed in relative terms, some small, specialized universities reveal themselves to be more efficient at producing HCPs (% of HCPs or citations per HCP). We think that this methodology, due to its simplicity, its ease of calculation, and the knowledge it provides, can be very useful to analyze the national systems of any country, in order to know the impact and visibility of the research carried out in its scientific institutions or research areas.

## Introduction

Right from the creation of the impact factor (IF) by Garfield (1955), the field of scientometrics assumed this indicator as a measure of analysis of scientific performance based on the number of citations a journal achieves. Thus, the impact of an author or an institution was held to be equal to the impact of the journals where their papers were published.

Although several studies have shown that journal's IF does not accurately reflect the impact of each individual article (Seglen, 1997; Garfield, 2006), many institutions and national evaluation systems still use it.

The debate over the use (and abuse) of the IF, and criticism of its application and reformulations, could be reduced if, as some authors explain, it is assumed that the IF has played a meritorious role in identifying influential journals and should continue to be used as an indicator of competitiveness and reputation. That is, as an indicator of the capacity of an author or institution to publish in journals with a high publication demand (Orduña-Malea et al., 2016).

With the appearance of Web 2.0. there has been an unprecedented change in the world of scientific activity production and dissemination. The use of new platforms to generate and share data and research results as well as the creation of digital identities have influenced the field of research evaluation (De Filippo and Sanz-Casado, 2018). Traditional studies based on bibliometrics can be complemented with new indicators such as altmetrics, which measure the interest that research arouses in society and have had a particular impact since their appearance in 2010 (Priem and Hemminger, 2010). One of the main advantages of these indicators is that, since the data are presented at the article level, a study's impact can be evaluated without considering the quality or visibility of the journal of publication (Neylon and Wu, 2009). As Martín-Martín et al. (2018) comment, “Since the classic study by Bollen et al. (2009), where the data came primarily from usage logs provided by publishers, many papers have been published on the nature of online article-level metrics.” Some of these studies have tried to correlate traditional citation with citation over different platforms that offer indicators of social media impact.

The possibility of analysis provided by new social media and platforms has led some authors (Orduña-Malea et al., 2016; Martín-Martín et al., 2018) to mention the emergence of a new line of bibliometric research, ALMetrics (author level metrics), which analyses the performance of authors by measuring all the dimensions of their intellectual activity. Without a doubt, many challenges arise with these options for assessment at the document and author level, not only from a technical point of view but also, and especially, for the study and evolution of impact and visibility.

From the point of view of research evaluation, both at individual and institutional level, another indicator that has started to be used in the last decades is highly cited papers (HCPs). One reasons for this is the increasing focus on scientific excellence in scientific policy (van-Raan, 2000). Science policy is increasingly interested in scientific excellence given its new public management tools (Aknes, 2003; Lamont, 2012). “Many countries are moving toward research policies that emphasize excellence; consequently; they develop evaluation systems to identify universities, research groups, and researchers that can be said to be “excellent” (Danell, 2011). This was shown in a diverse studies as a benchmarking study from the European Commission in which HCPs were used as indicators for comparing the research performance of the EU countries (European Commission, 2001). Highly cited papers have also been applied as indicators in case studies of research groups and some authors concluded that highly cited research papers do represent useful indicators for identifying “worldclass” research (Tijssen et al., 2002).

In recent years, the use of highly cited articles has become increasingly common and indicators, such as those developed by Clarivate Analytics (2020), are being widely used for institutional evaluation. In the field of higher education, indicators of excellence in research have also been developed, such as those offered by the Ranking of SCImago Institutions (Bornmann et al., 2012), the mapping of excellence (Bornmann et al., 2014), and the Ranking Leiden (Waltman et al., 2012). Despite the increasingly frequent use of these indicators, these indices are not exempt from criticism, both from the methodological point of view and their application (Hu et al., 2018), so it is essential to continue developing the research in this field.

In this line, some authors also mention that it is urgent to look further into the phenomenon of HCPs, especially in small and peripheral countries, where the need to be selective is largest, the citation indicator is more uncertain than in core countries (Aknes, 2003).

In this context, highly cited articles have been considered as potential candidates for identifying and monitoring “excellent” scientific research. A wide range of options lies open for the analysis of the scientific activity of institutions such as universities, one of the main producers of knowledge, whose evaluation requires precise tools.

### Institutional-Level Metrics: Evaluation in Universities

The analysis and evaluation of the research activity carried out in the institutions has been a decisive step to really know the scope of these activities, make proposals, and offer society the necessary transparency of its efficient management of the resources allocated to the research carried out in these institutions. In this way, Szomszor et al. (2021) state that “Research evaluation may be seen as a reflection of a broader societal shift to institutional managerialism and public sector accountability.” However, it is within higher education institutions where evaluation has been more ingrained and where it is playing a more decisive role. The reasons why this effects have occurred are several, for example, accountability to society for the activities they carry out, the proper management of the financial resources they receive, or knowing how the scientific productivity of their academic staff evolves. One of the countries that first considered the need to evaluate its higher education institutions was the United Kingdom, where the first national Research Selectivity Exercise was introduced in 1986 and led to a more formalized and structured Research Assessment Exercise (RAE) from 1992 (Szomszor et al., 2021). This evaluation process has currently changed its name to Research Excellence Framework (REF) (REF, 2020) and it has had multiple counterparts in different countries (Sanz-Casado et al., 2013), especially in the Nordic countries (Sivertsen, 2018) and in Australia where the Australian Research Council (ARC) conducted the first Excellence in Research for Australia (ERA) evaluation in 2010 (ARC, 2019). These institutional evaluation processes have gained renewed importance with the emergence of international university rankings since 2003.

Rankings such as the Shanghai (ARWU), Times Higher Education (THE), and QS have had great impact, and they have served to provide information on higher education institutions around the world. These rankings, which have spurred the debate about the quality and performance of higher education systems, have had a considerable impact on our global society in light of the internationalization of higher education. That, in turn, has heightened global competition and induced proliferation of this type of studies (De Filippo et al., 2012). However, criticism of their methodology and implementation has also been plentiful (Liu and Cheng, 2005; van-Raan, 2005; Buesa et al., 2009). The methodology used to formulate these rankings cannot deliver reliable data for more than 700–1,200 institutions, in the light of the wide range of variation in the non-top-ranked universities. Another frequent criticism is that this methodology may therefore be regarded as “elitist,” inasmuch as it entails excluding the vast majority of the world's universities (De Filippo et al., 2012).

The need to complement the information provided by international classifications has fostered the development of some initiatives with data at the national level. Several rankings have been developed in Spain, such as the Multidimensional Index of University Quality (Buesa et al., 2009), the Research Ranking of Spanish Public Universities (Buela-Casal et al., 2011), the General and Area Ranking of Spanish University Institutions (Corera et al., 2010), the I-UGR Ranking of Spanish Universities (Torres-Salinas et al., 2011), and the Observatory of Research Activity of the Spanish University (IUNE) with annual updates from 2012 (Sanz-Casado et al., 2011, 2013; De Filippo et al., 2014).

The IUNE Observatory, created by the 4 Universities Alliance (A4U), has the support of the Spanish Ministry of Universities and offers aggregate information on seven dimensions (teaching staff, scientific recognition, scientific activity, innovation, research training capacity, competitiveness, and funding). The data are obtained from official and public sources and are presented through 48 indicators. The scientific publications are collected from the Web of Science core collection (www.iune.es).

One of IUNE's basic premises is the presentation of a wide range of indicators to present a simple, transparent picture of each institution's scientific activity, trying to account for the variety of profiles in existence. This is possible because IUNE considers a large number of indicators related to the scientific and knowledge transfer activity of Spanish universities, unlike other rankings that assign the greatest weight to bibliometric indicators.

The great variety of data obtained enables basic information to be displayed over the web by university, by major fields of knowledge and in terms of the university system as a whole (Bautista-Puig et al., 2020). Among the indicators within the IUNE framework, different metrics are being developed that consider the document as the object of study. Some of them, related to impact, are presented below. The calculation of indicators at the local (country) level is key to making comparisons between institutions in the same context. In this paper we present the methodology developed to calculate HCPs without using international comparison that may be far removed from local practices.

## Objectives

The research presented in this paper has been aimed at the following objectives:

- To develop a methodological proposal based on the identification of HCPs in domestic systems, such as the Spanish Public University System (SUPE), in order to find the most outstanding publications in the Spanish scientific context.
- To detect different activity and impact profiles among Spanish universities. This will make it possible to differentiate those universities that play a more significant role in these two important aspects.

## Methodology

This study uses a specific methodology to explore each Spanish public university's highly cited papers at the domestic level (HCP-DL), calculated in relation to the total scientific production of the SUPE in the Web of Science citation indexes.

The data obtained from the IUNE Observatory, which includes publications collected from the three main databases of the Web of Science core collection (Science Citation Index, Social Science Citation Index, Arts and Humanities Citation Index), are used as a source of information.

To identify the SUPE's production, a system based on regular expressions is used to encode and normalize the signature of each document. Regular expressions are patterns used to find a certain combination of characters within a text string (Ruslan, 2003). This enables each university's publications to be identified by searching for different signature variants in the “address” field. This system assigns publications to each institution using the total count of documents (one publication is counted for each signatory institution). Although there are standardization options such as the “enhanced organization” of Web of Science, the identification by regular expressions, which has been used at IUNE for more than a decade, has different advantages. On the one hand, it allows a “strict” attribution of documents, i.e., it only considers the university's own production (not including documents produced by university hospitals, health centers, consortia, etc., in which the explicit signature of a university does not appear). It also allows information to be retrieved from incomplete signatures (only postal addresses, names of centers or departments, which clearly belong to a university). With this system, some universities see an increase in their output compared to the direct WoS query (as greater flexibility and breadth in the identification of university documents is possible), while others see a reduction in their output (by eliminating documents considered to be “university documents” but without an explicit signature). APPENDIX I (of the Supplementary Material) provides a comparative table retrieving information from WoS and IUNE. The use of regular expressions for the identification of the production of one university (Universidad Carlos III de Madrid) is also presented.

The Spanish university system currently has 83 universities (50 public and 33 private). This study analyses the production of public universities since they have more-intensive research activity and produce more than 95% of the Spanish university system's publications (Casani et al., 2013). The list of public universities and their acronyms is shown in IUNE Glosario (IUNE, 2020).

The study is carried out per year, and the analysis period includes the publications of 2014–2017 (citations were collected in February 2020). This citation window has been chosen so that publications have at least 2 years of citations; otherwise, the data may be very distorted. The study has been carried out considering the production by subject area, given that there are differences in the dynamics of production, impact, and visibility of the different scientific disciplines (Aknes, 2003; Aksnes and Sivertsen, 2004). For this purpose, the thematic aggregation carried out by Web of Science (Incites/Essential Science Indicators areas) was considered. Twenty-two areas were considered, plus one more area, humanities, to differentiate the production of this field from that of Social Sciences, which has important differences (Huang and Chang, 2008). Lists were thus produced for each year and area, with the publications ordered by the number of citations received. This process allows us to determine the minimum number of citations that a publication must have obtained in order to be considered a HCP.

The first relevant information to find is the distribution of citations by thematic area in the SUPE. The publications were classified into different groups according to their impact. There are various definitions of what counts as a highly cited article. Basically two different approaches can be identified, involving absolute, or relative thresholds (Aknes, 2003). Therefore, in this study the publications have been classified into three groups: (i) Un-citedness (documents without citations up to the time of data collection); (ii) less-citedness (documents receiving between 1 and 10 citations); (iii) most-citedness (documents receiving more than 100 citations). These limits were established for convenience and for simplicity of comprehension. This calculation provides information on the general dynamics of citation in the SUPE.

Next, the first step for calculating HCP-DL is to determine which and how many are the HCP-DL that are collected in the Web of Science, establishing three citation thresholds (1, 5, and 10%). Once the papers are ranked, the number of citations that a publication needs to be considered HCP-DL is selected.

Some indicators are calculated from this data:

- number of HCP-DL at public Spanish universities and by InCite area.
- percentage of highly cited papers (HCP-DL) for each university.
- average number of citations per document received by HCP-DL.

**Figure 1:**
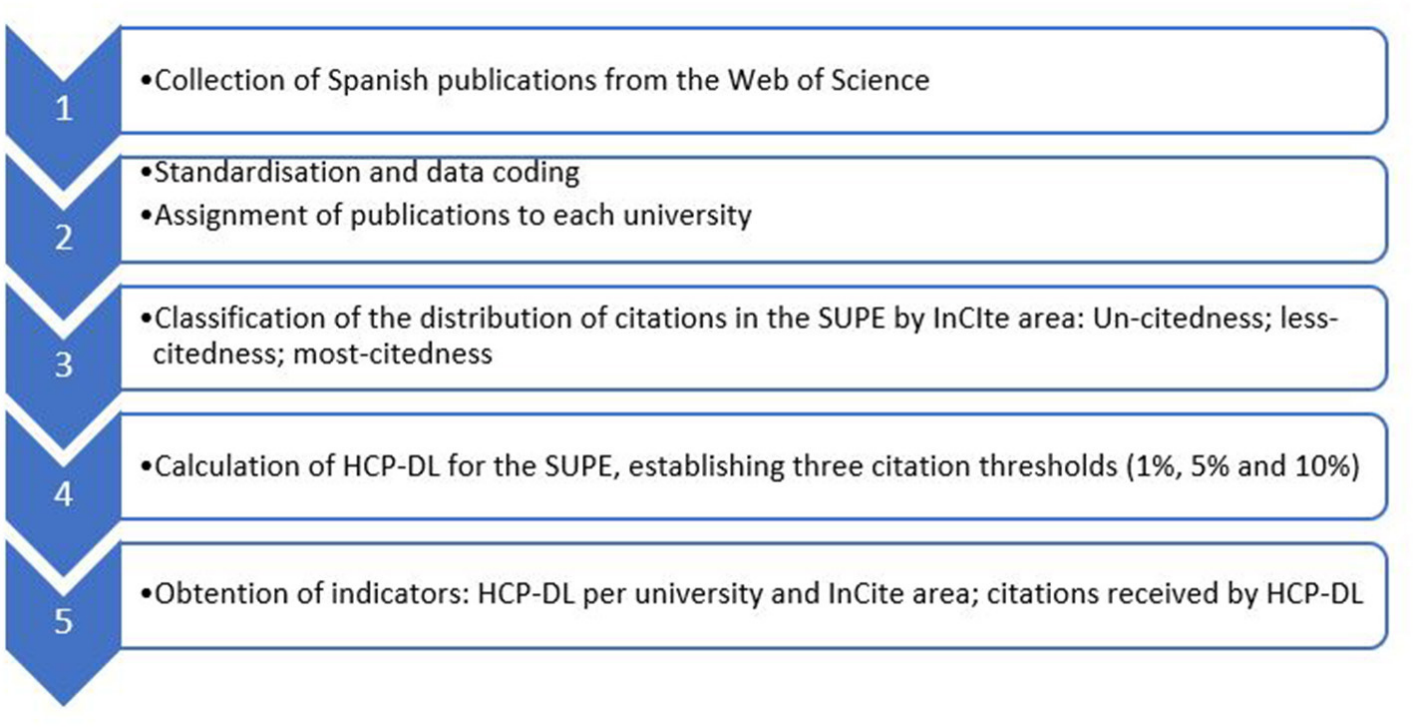
Methodological steps.

Figure 1 shows the methodological steps followed.

## Results

From 2014 to 2017 the SUPE published 218,779 documents in the Web of Science core collection. The main results of each phase of the HCP-DL calculation process are presented below.

### Citedness Distribution

**Figure 2:**
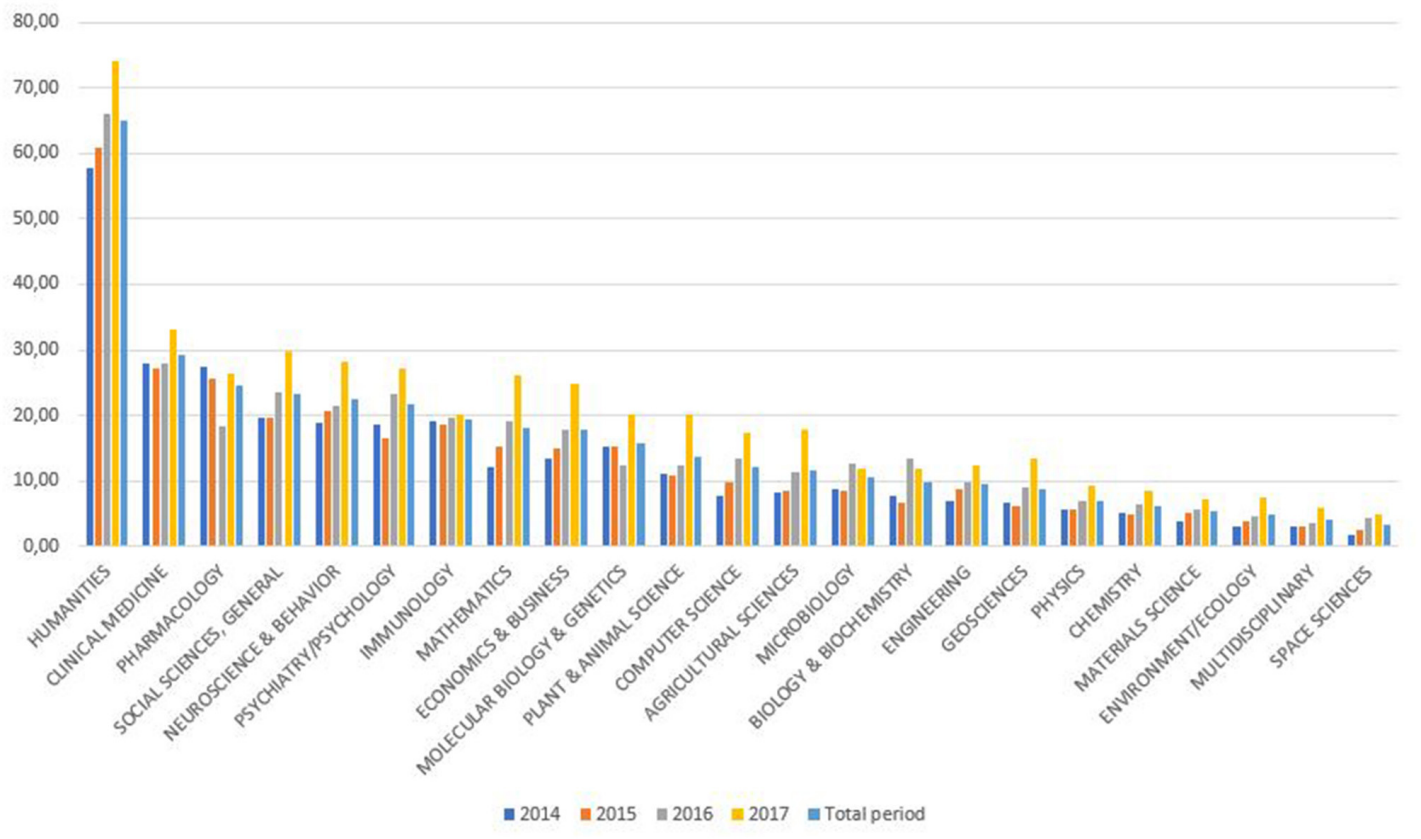
Percentage of un-citedness in SUPE by Incites/Essential Science Indicators area.

**Figure 3:**
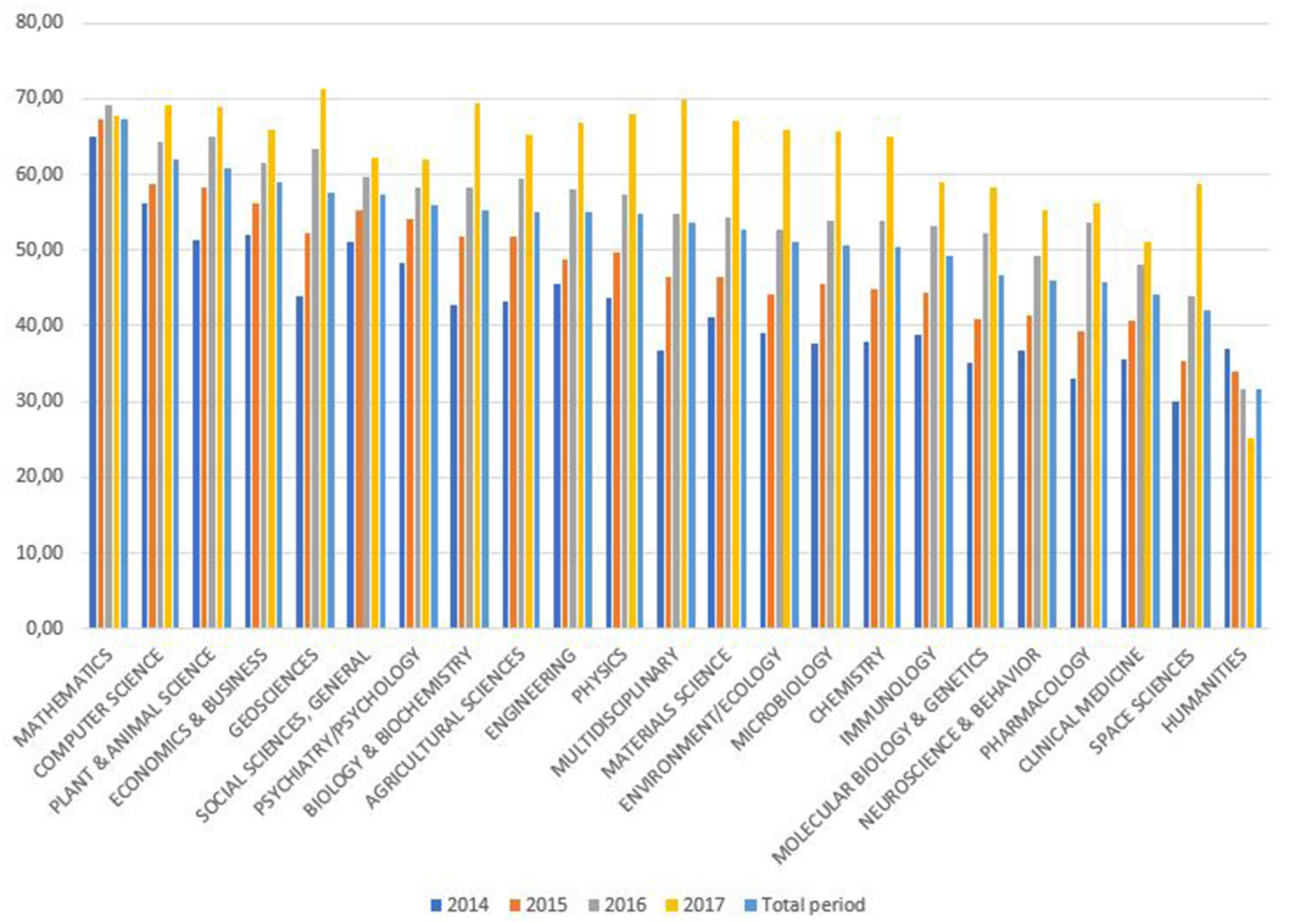
Percentage of less-citedness in SUPE by Incites/Essential Science Indicators area.

**Figure 4:**
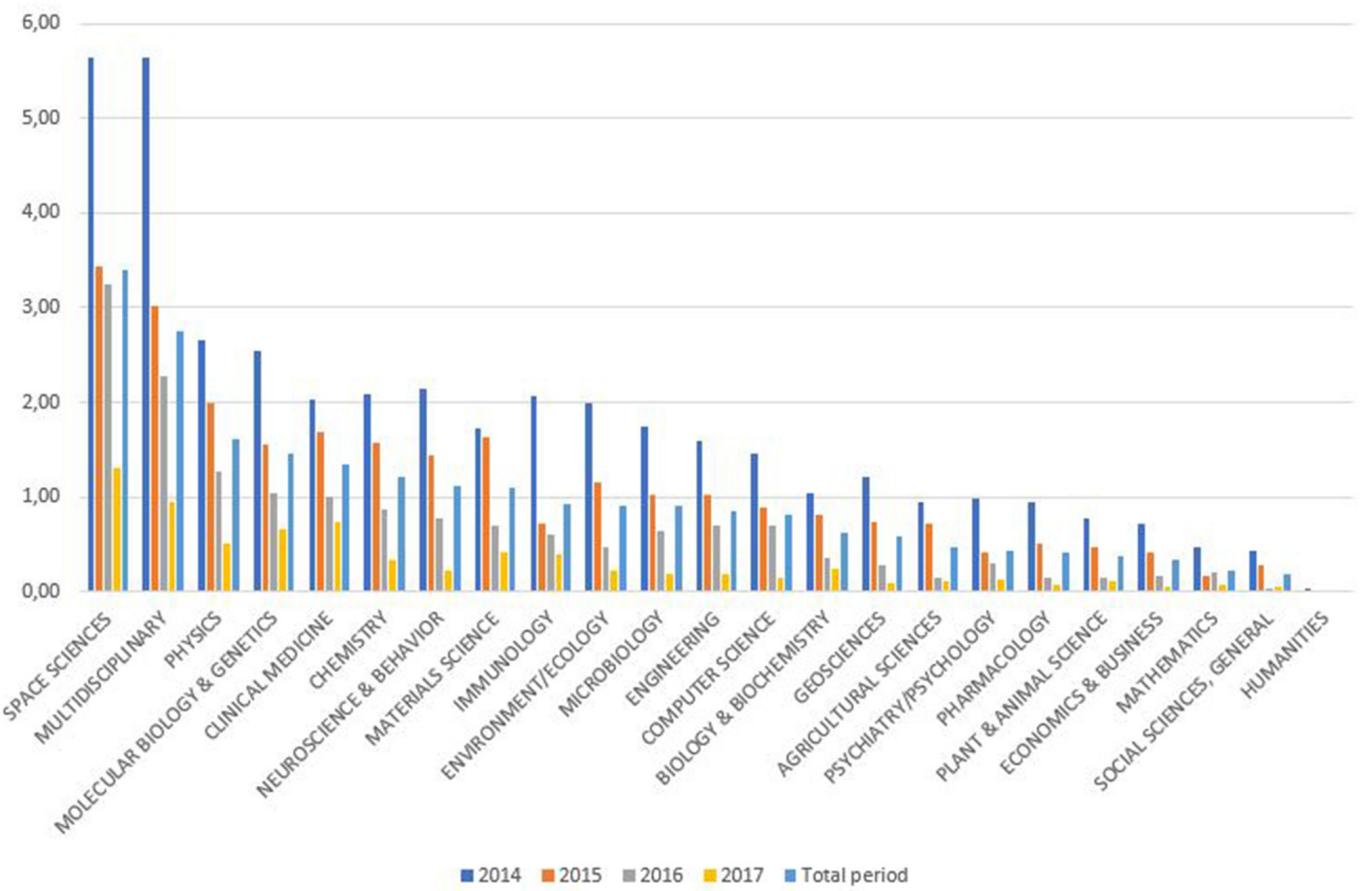
Percentage of most-citedness in SUPE by Incites/Essential Science Indicators area.

Figures 2–**4** show the distribution of citedness on three levels. The data is sorted in descending order by the total number of documents for each subject area in the period. Tables with the corresponding percentage values are given in APPENDIX II (Supplementary Material). The percentage of documents not cited is presented in Figure 2, distributed by thematic area and year. Humanities is the field with the highest proportion of uncited documents (65%). Other areas, such as Clinical Medicine, Neurosciences, Pharmacology, Mathematics, Immunology, and Molecular Biology, also have high un-citedness percentages. On the other hand, Space Science only has 3.4% non-cited documents and is the area with the highest visibility.

The proportions of less-cited documents receiving between 1 and 10 citations are similar in most areas, with a percentage range of between 50 and 60% (Figure 3). The area with the lowest proportion of less-cited documents is Humanities (31.8%), and Mathematics has the highest average (67.3%).

Figure 4 shows the documents with more than 100 citations, which reach higher levels only in the areas of Space Science (3.4%) and, to a lesser extent, Multidisciplinary (2.76%).

### Highly Cited Papers at the Domestic Level

This section presents the distribution of citations received by documents published by SUPE universities, establishing three dynamic thresholds that vary according to the year of publication and the Incites/Essential Science Indicators area in which the journal is classified.

In order to define the conditions a paper must fulfill to be considered an HCP-DL, a minimum citation threshold is established, by area and year, for the top 1, 5, and 10% of the most-cited documents. These limits are shown in Table 1, where, for example, an article published in 2014 in Space Sciences needs 376 citations (or more) in 2020 to place among the top 1% of the most-cited documents in its area, but it needs only 124 citations if it is published in 2017. The total number of HCP-DLs by area for the 4-year period is shown in APPENDIX III (included as Supplementary Material).

**Table 1:** HCP-DL lower limit, minimum number of citations needed to qualify as HCP-DL.

**Incites/Essential science indicators area**	**1%**				**5%**				**10%**			
	**2014**	**2015**	**2016**	**2017**	**2014**	**2015**	**2016**	**2017**	**2014**	**2015**	**2016**	**2017**
Agricultural Sciences	96	84	52	41	48	39	29	21	35	29	21	15
Biology and Biochemistry	101	92	67	48	51	41	30	22	36	27	20	15
Chemistry	157	129	89	55	62	53	40	27	43	36	28	19
Clinical Medicine	146	131	100	77	56	48	32	25	35	29	21	15
Computer Science	122	97	71	47	48	36	27	19	30	25	18	12
Economics and Business	94	70	49	36	49	33	26	15	33	24	17	10
Engineering	122	101	80	49	56	46	34	24	38	32	24	17
Environment/Ecology	152	111	81	54	62	53	41	27	43	39	29	20
Geosciences	115	91	67	38	47	40	30	19	34	27	20	13
Humanities	26	21	15	9	11	11	6	4	6	5	4	2
Immunology	150	84	82	53	58	44	34	26	40	28	22	18
Materials Science	122	125	85	60	60	52	38	29	40	37	28	20
Mathematics	63	50	33	25	28	22	17	12	19	15	12	8
Microbiology	132	103	78	59	58	44	34	26	39	32	23	17
Molecular Biology and Genetics	159	126	108	68	64	51	38	27	42	33	26	17
Multidisciplinary	326	175	170	98	115	68	59	31	62	44	36	20
Neuroscience and Behavior	132	143	85	47	59	46	37	22	40	30	24	14
Pharmacology	99	71	55	37	45	38	31	21	30	27	21	14
Physics	184	148	124	73	68	55	41	28	45	37	27	18
Plant and Animal Science	90	75	51	34	41	34	24	16	29	22	17	11
Psychiatry/Psychology	101	69	53	36	45	33	25	17	29	22	16	11
Social Sciences. General	73	60	44	29	38	29	21	14	25	21	15	9
Space Sciences	376	224	231	124	110	85	68	44	71	54	45	27

The distribution of HCP-DLs by Incites/Essential Science Indicators area in the SUPE universities is presented below, using the limits established in Table 1. Table 2 shows the first 10 universities by absolute number of HCPs in all three top citation groups. This table represents the absolute numbers of HCP-DL for each institution by research area. The values have been colored with a gradient ranging from green (the universities with the highest HCP-DL score) to red (those with the lowest HCP-DL score). The positions change little. The size of universities was determined in base of their number of students (QS Intelligence Unite, 2020) and it can be observed that que the biggest universities (UB, UAB, UV, UAM, UGR, UCM) occupy the leading positions in all three top citation groups. Interestingly, one small university (UPF) places seventh and eighth in the two most-demanding groups. The absolute values for all public universities and Incites/Essential Science Indicators areas are shown in APPENDIX IV (Supplementary Material).

**Table 2:**
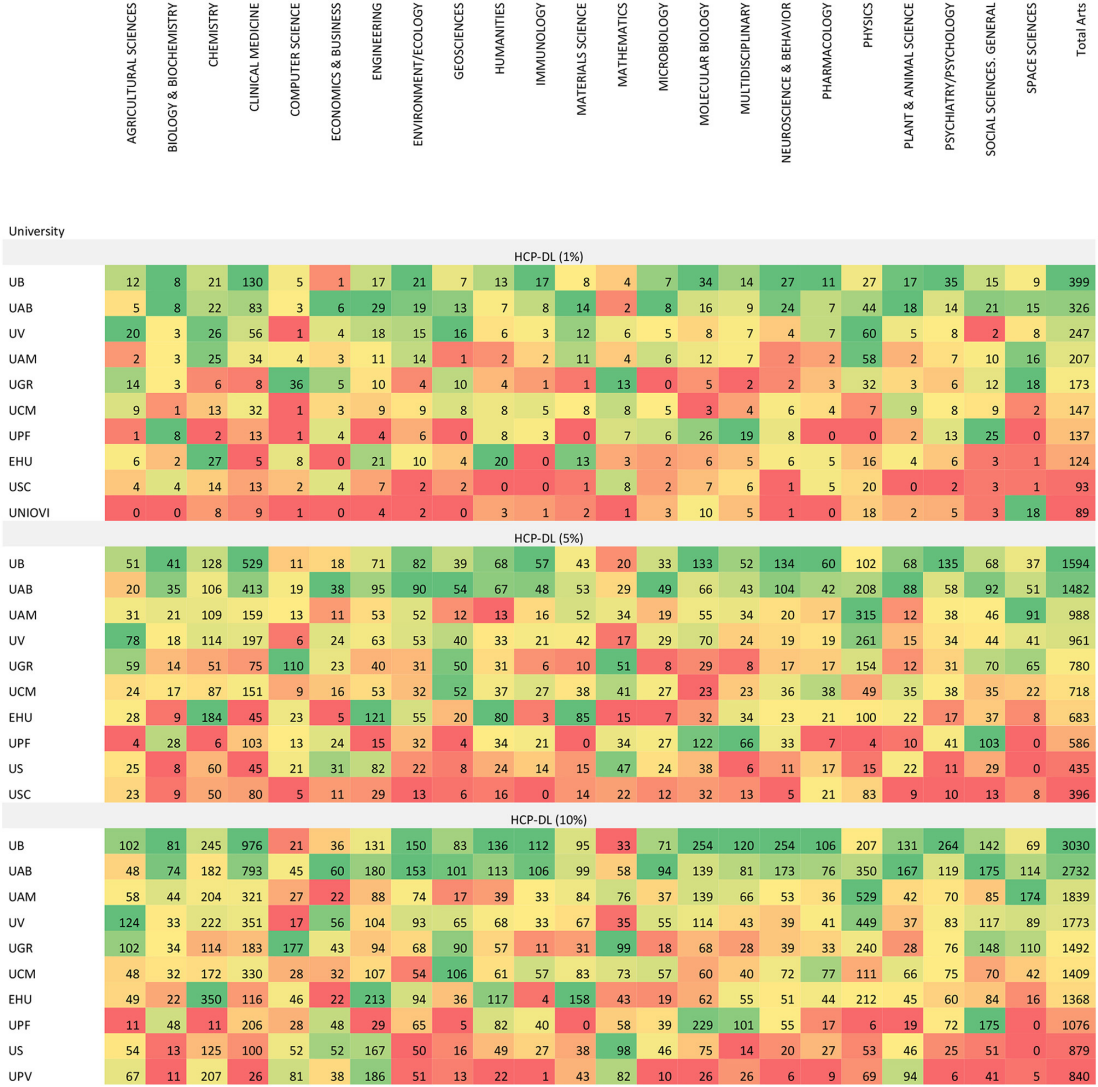
HCP-DLs by university and incites/essential science indicators area in all three top citation groups (absolute values).

*Color shades indicates the magnitude (green are universities and subject areas with higher values and red with lower values)*.

The percentage of HCPs by university and area has also been calculated. The values for the first 10 universities by percentages are shown in Table 3. The proportions do not always follow the same order as the absolute values. The percentage values by area for all universities are presented in APPENDIX V (Supplementary Material).

**Table 3:**
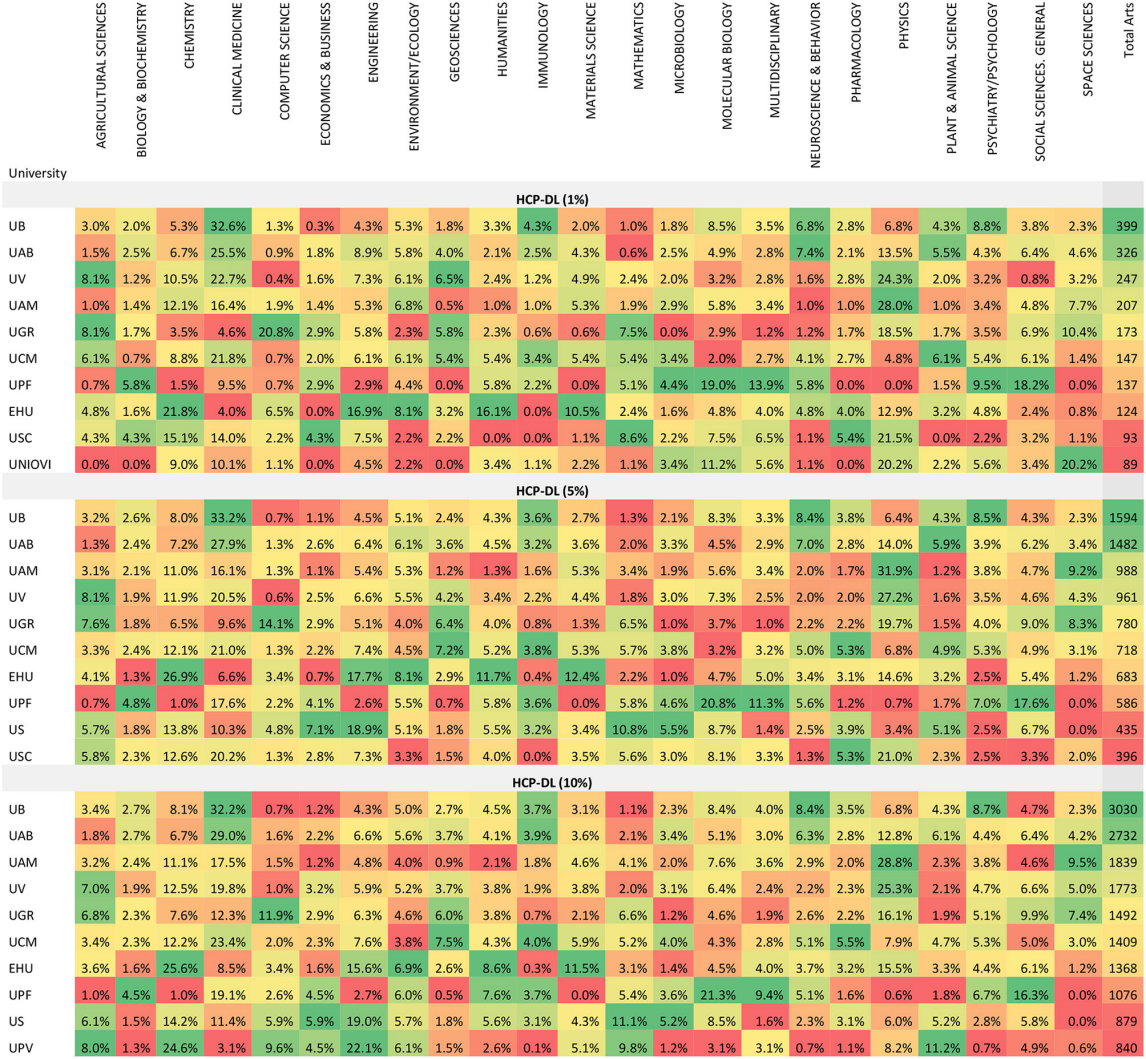
HCP-DLs by university and incites/essential science indicators area in all three top citation groups (percentages).

*Color shades indicates the magnitude (green are universities and subject areas with higher values and red with lower values)*.

Once the values for each area are calculated, the total number of HCP-DLs for each university can be found. Table 4 shows the number of documents that exceed the citation limits at each institution as well as the publishing effort, measured as the percentage of publications that have crossed the citation thresholds out of the total number of documents produced by the university. The last columns show the position (rank) of each institution by its number of documents in the top 1% and by its percentage in the top 1%, together with the changes of position of each institution in terms of rankings and in terms of each citation threshold.

**Table 4:**
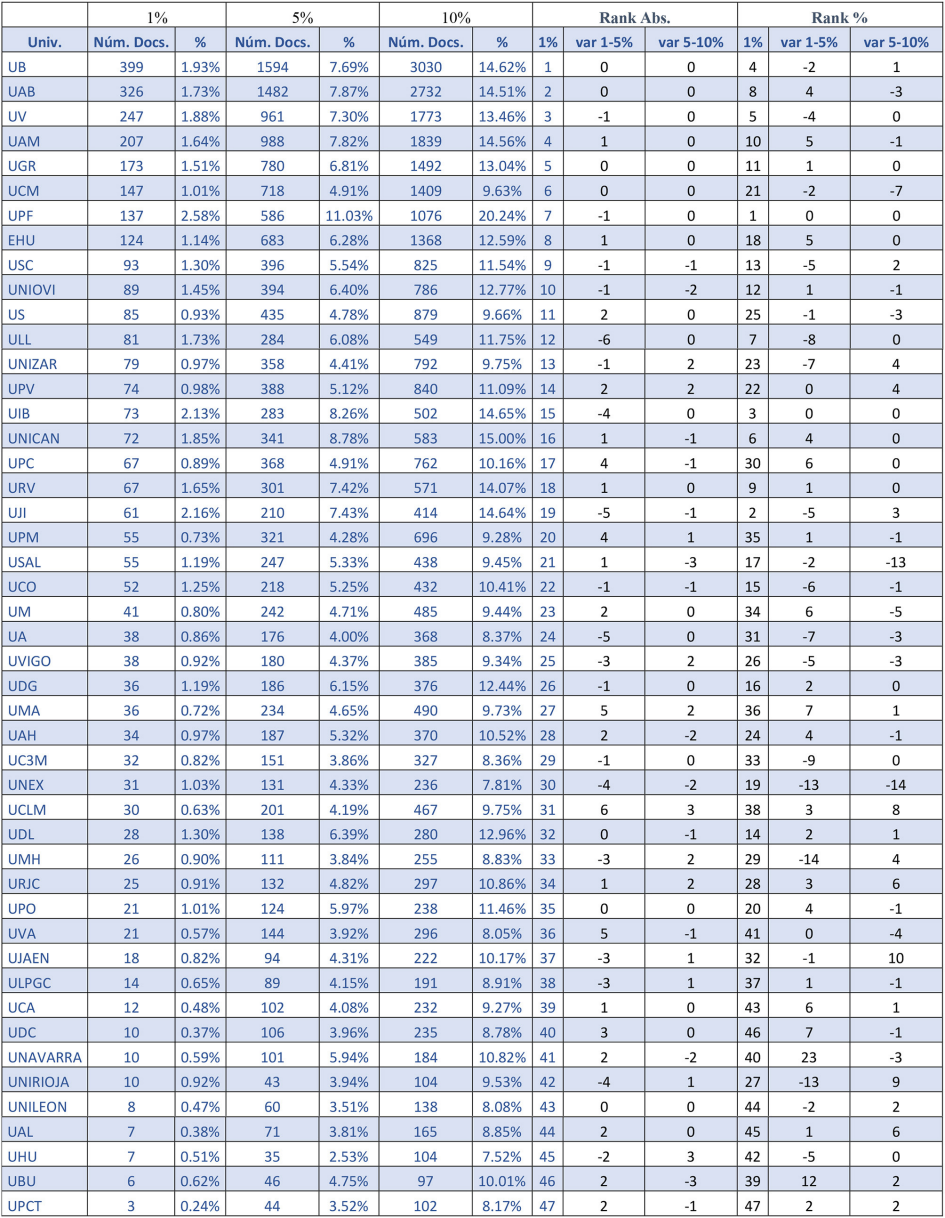
Number and percentage of HCP-DL documents by university (ordered by absolute number of HCP-DL documents in the top 1%).

According to the data in Table 4, the leading positions are occupied by the large universities, and their order by number of documents remains practically unchanged in the first 10 positions. However, the positions change drastically when publishing efforts (percentage of documents) are compared. With the exception of UPF, which presents the highest HCP-DL percentages in all three top groups, the rest of the universities positioned in the first 10 by number of highly cited documents fall to positions ranging from fourth place for UB to 21st place for UCM.

**Figure 5:**
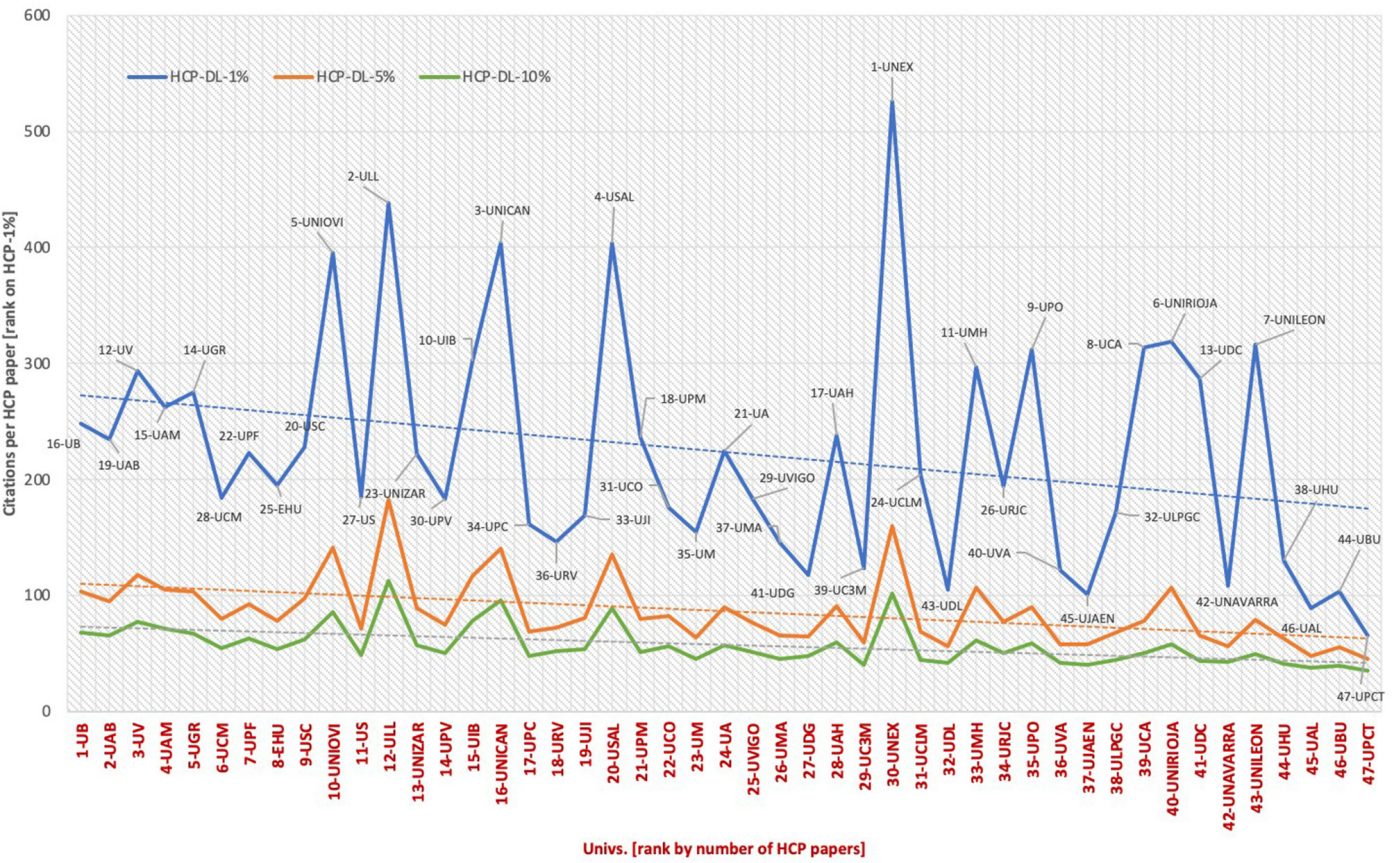
HCP-DL percentage at Spanish public universities (1, 5, and 10%).

Figure 5 shows the HCP-DL ratios for public universities at the three citation levels. The universities are presented in order by total number of documents. The positions by publication effort for the top 10% citation group are also presented.

### Citations per HCP

**Figure 6:**
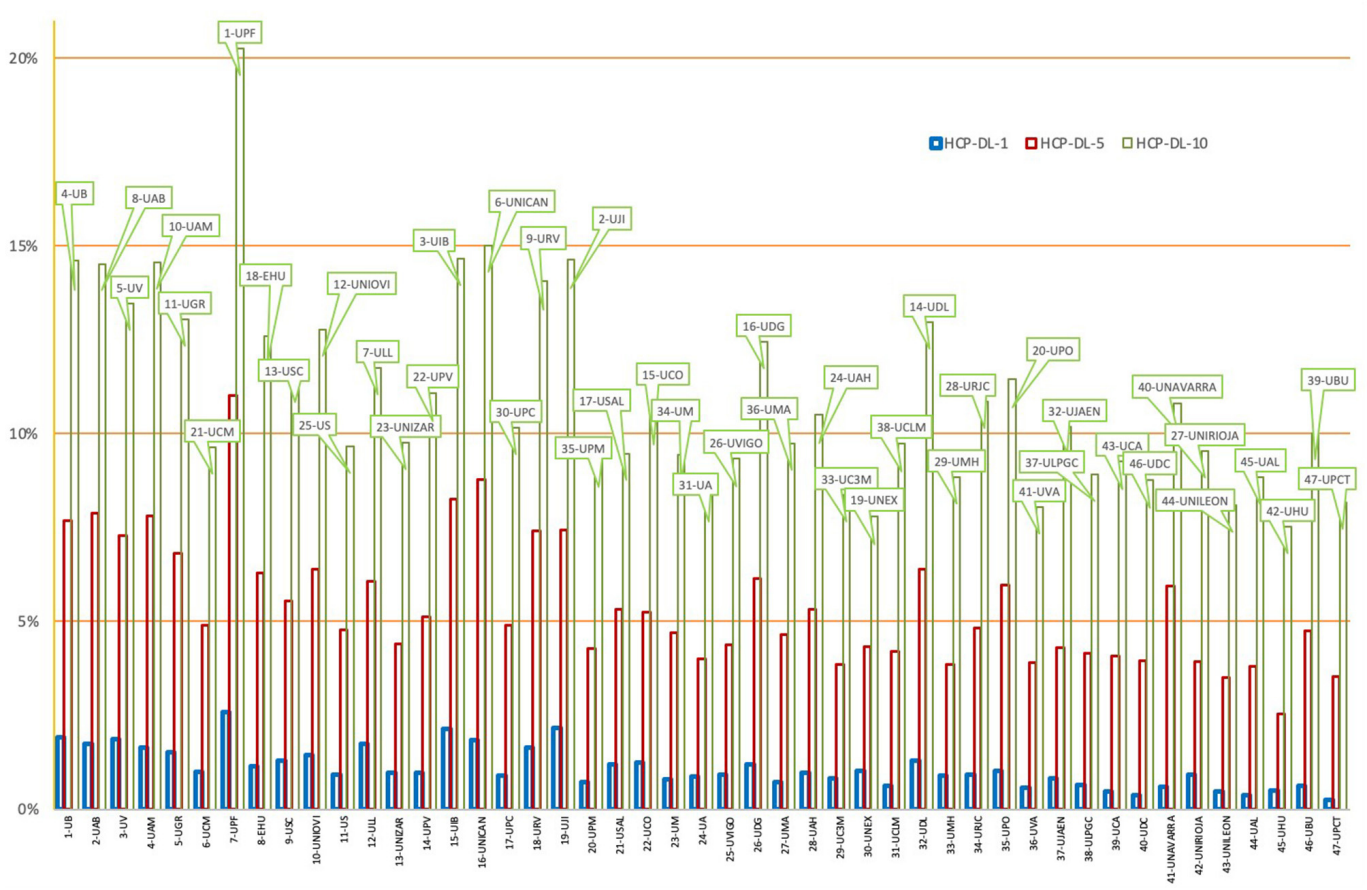
Observed averages of citations per HCP compared to period average thresholds.

Once the HCP-DLs have been calculated, a relationship can be found between HCP-DLs and the number of citations received. Figure 6 shows the comparison between the average number of citations per SUPE document in each Incites/Essential Science Indicators area (Observed averages) and the average per area of the annual thresholds (Threshold average). The data show that the highest averages are in Space Sciences and that Clinical Medicine is in fourth place ahead of Physics.

In addition to revealing the characteristics of the SUPE, these data enable comparisons to be drawn with the information extracted from the WoS HCPs indicator. Although caution is required due to possible differences in the analysis periods or citation windows, some differences can be found. In the case of SUPE, for HCP-DL 1% the thresholds are much higher in Space Sciences (i.e., WoS: 157^1^ in 2014, SUPE: 376; or in averages: 120 vs. 238.8) and Multidisciplinary (196 vs. 326), while in Clinical Medicine the average is slightly higher in the case of SUPE than WoS (100.8 vs. 113.5).

**Figure 7:**
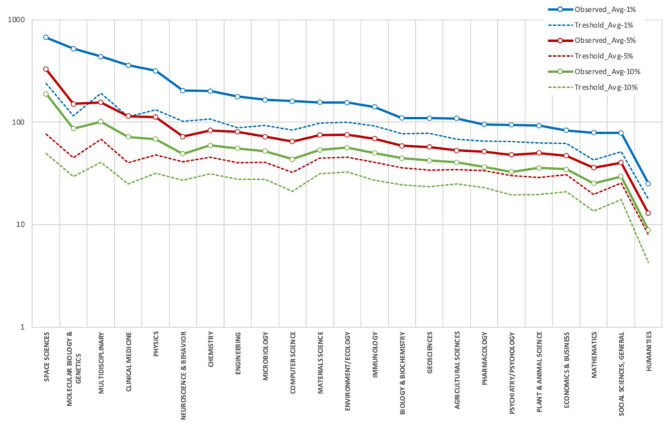
Citation per document by university 2014–2017.

By going down to the university level, the number of HCP-DLs can be compared with the citations per HCP document at each institution in the three top citation groups. Figure 7 shows this relationship by presenting the universities ordered on the abscissa axis by the number of HCP-1% documents (this value appears on the label). To the right of the figure are positioned the universities with the lowest HCP volume, and to the left, those with the highest. Above the trend lines are the universities with higher than expected value in terms of citations per HCP document.

## Discussion and Conclusions

The present study proposes a methodology focused on identifying HCPs produced by Spanish public universities using the figures for the SUPE as a reference. The proposed indicator, which is termed Highly Cited Paper at the Domestic Level (HCP-DL), provides a new context of comparison that is much better for comparing universities in the same system than the indicators offered by the Web of Science, whose reference is publications world-wide, because the HCP-DL considers the real citation values of the documents published by institutions in the same country.

A number of methodological considerations should be borne in mind. First, to obtain results such as those presented in this paper, there must be a citation window of at least 2 years, to ensure that the citation values of the most-recent publications are consistent with those of the rest of the period. Newly published papers must be allowed time to be cited. That is the reason why the documents analyzed here were published between 2014 and 2017, with citations gathered in February 2020. Future plans are for this methodology to be used to analyse consecutive periods with moving citation windows (2015–2018; 2016–2019; 2017–2020) and thus analyse the development of HCPs by university and by subject area. In addition, the method is easy to use as an additional indicator for evaluating systems like the SUPE due to its relative ease of calculation (as world reference figures are not needed).

Another point is that the comparison makes sense only in the framework of well-defined subject areas. Although Incites/Essential Science Indicators categories have proved adequate, we have included the area of Humanities, which has characteristics of its own; and we believe it is important to differentiate Humanities from Social Sciences, since Humanities has specific citation characteristics that differ from those of many of the social science disciplines. In this way, other authors (Hellqvist, 2010; McManus and Neves, 2020) find that databases such as the Web of Science are too narrow in scope, humanistic scholars publish in their native language and not in English-language journals, and they publish in monographs and anthologies rather than journals he humanities scholars. Another characteristic that also differentiates these researchers from social scientists is that they produce a greater variety of publications, value books, study topics of regional and cultural concerns, and cite much older literature (Huang and Chang, 2008). Therefore, we recommend using this criterion.

The results of the case study of the Spanish university system show a preponderance of HCPs in the field of Space Science. This is because of a tightly clustered small number of Spanish institutions that are members of major international cooperation networks in the category of Astronomy and Astrophysics and publish accordingly. The major non-specialized universities (e.g., UB, UAB, UAM, UGR, UV, UCM) are also observed to have HCPs in many areas, while the polytechnic universities have high visibility in the Computer Science area. It has been observed generally that the presence of HCPs in a given area has to do with a university's teaching and research specialities. For example, UC3M presents domestic HCPs in Economics, Engineering, and Mathematics, but not in areas not covered by its teaching plan, such as the medical sciences.

Obviously, in absolute terms, as the findings of this paper have shown, the big non-specialized universities are major producers of HCPs and hold the leading positions in our results. However, when efficiency is analyzed in relative terms, some small, universities (like UPF, UC3M, UNIOVI, ULL, and UIB) reveal themselves to be more efficient at producing HCPs (% of HCPs or citations per HCP). Big universities like UB, UAB, UV, UGR, and UAM are also highly efficient and have high HCP percentages in certain categories. Furthermore, there is a large number of universities in the SUPE whose HCP numbers, both absolute and relative, are quite remote from those of the universities mentioned above.

The interest this study has aroused in policymakers, scientific and academic authorities and Spanish accreditation agencies has led us to present the methodology in this special issue on good practices. We believe that, because of its simplicity, its ease of calculation and the knowledge it provides, it can be exported to analyse any country's national systems with a view to ascertaining the impact and visibility of the research done in that country's scientific institutions or in their research subject areas.

## Data Availability Statement

The original contributions presented in the study are included in the article/Supplementary Material, further inquiries can be directed to the corresponding author/s.

## Author Contributions

CG-Z: design and implementation of the research, methodological development, data analysis, visualization of data, and writing of the manuscript. SM: computer and methodological development and data extraction. DD: data analysis, writing of the manuscript, and funding acquisition. ES-C: discussion of results, revision of data, and revision of manuscript. All authors contributed to the article and approved the submitted version.

## Conflict of Interest

The authors declare that the research was conducted in the absence of any commercial or financial relationships that could be construed as a potential conflict of interest.

## Footnotes

^1^InCItes Essential Science Indicators. Highly Cited Threshold https://esi.clarivate.com/ThresholdsAction.action.
